# The effect of transcranial electrical stimulation on the relief of mental fatigue

**DOI:** 10.3389/fnins.2024.1359446

**Published:** 2024-06-17

**Authors:** Ruijuan Chen, Lengjie Huang, Rui Wang, Jieying Fei, Huiquan Wang, Jinhai Wang

**Affiliations:** ^1^School of Life Sciences, Tiangong University, Tianjin, China; ^2^School of Electronics and Information Engineering, Tiangong University, Tianjin, China

**Keywords:** mental fatigue, tDCS, α-tACS, ECG, HRV

## Abstract

**Objective:**

The presence of mental fatigue seriously affects daily life and working conditions. Non-invasive transcranial electrical stimulation has become an increasingly popular tool for relieving mental fatigue. We investigated whether transcranial direct current stimulation (tDCS) and transcranial alternating current stimulation (tACS) could be used to alleviate the state of mental fatigue in a population of healthy young adults and compared their effects.

**Methods:**

We recruited 10 participants for a blank control, repeated measures study. Each participant received 15 min of anodal tDCS, α-tACS, and blank stimulation. Participants were required to fill in the scale, perform the test task and collect ECG signals in the baseline, fatigue and post-stimulus states. We then assessed participants’ subjective fatigue scale scores, test task accuracy and HRV characteristics of ECG signals separately.

**Results:**

We found that both anodal tDCS and α-tACS significantly (*P* < 0.05) reduced subjective fatigue and improved accuracy on the test task compared to the blank group, and the extent of change was greater with tACS. For the HRV features extracted from ECG signals. After tACS intervention, SDNN (*t* = −3.241, *P* = 0.002), LF (*t* = −3.511, *P* = 0.001), LFn (*t* = −3.122, *P* = 0.002), LFn/HFn (−2.928, *P* = 0.005), TP (*t* = −2.706, *P* = 0.008), VLF (*t* = −3.002, *P* = 0.004), SD2 (*t* = −3.594, *P* = 0.001) and VLI (*t* = −3.564, *P* = 0.001) showed a significant increasing trend, and HFn (*t* = 3.122, *P* = 0.002), SD1/SD2 (*t* = 3.158, *P* = 0.002) and CCM_1 (*t* = 3.106, *P* = 0.003) showed a significant decreasing trend. After tDCS intervention, only one feature, TINN, showed a significant upward trend (*P* < 0.05). The other features showed non-significant changes but roughly the same trend as the tACS group.

**Conclusion:**

Both tDCS and α-tACS can be effective in relieving mental fatigue, and α-tACS is more effective than tDCS. This study provides theoretical support for tDCS with α-tACS having a alleviating effect on mental fatigue and the use of ECG as a valid objective assessment tool.

## 1 Introduction

With the development of modern technology, people’s pace of life is getting faster and faster, and the pressure of work and competition has also increased. Prolonged exposure to intense work pressure, social pressure and family pressure, coupled with factors such as information overload and prolonged use of electronic devices, may lead to the emergence of mental fatigue. Mental fatigue has become an increasingly common state in today’s social life, and excessive mental fatigue not only affects daily life and work status (Van Cutsem et al., 2017; Holgado et al., 2021), but may also lead to cardiovascular and other diseases (Gecaite-Stonciene et al., 2021). Therefore, timely and effective interventions are needed to improve mental fatigue status. At present, there are many interventions to improve fatigue, such as caffeine (Herden and Weissert, 2020), odor stimulation (Wang et al., 2023), music (Gao et al., 2023) and outdoor activities (Abdollahzade et al., 2023). Nonetheless, these proposed strategies to counteract mental fatigue may not be readily implemented or effective in a timely manner. In recent years, noninvasive transcranial electrical stimulation (tES) has become an increasingly popular tool for the relief of mental fatigue. It is a technique that influences brain activity by applying electrical currents to the surface of the scalp and adjusting the excitatory, inhibitory, or synaptic transmission of neurons. Common non-invasive transcranial electrical stimulation techniques include transcranial direct current stimulation (tDCS), transcranial alternating current stimulation (tACS), and so on (Antal et al., 2017).

tDCS is a non-invasive technique that uses a weak direct current (usually 1–2 mA) passed between electrodes on the scalp to alter the neuronal membrane resting potential in a polarity-dependent manner, increasing or decreasing neuronal excitability in a given area (Sehm et al., 2013). This technique has been shown to have positive effects on alertness, working memory, and attention. For example, Dai et al. (2022) applied 1.5 mA anodal tDCS to the left dorsolateral prefrontal cortex (DLPFC) to enhance the early stages of relevant information processing and improve alertness, and Fernández et al. (2021) demonstrated that anodal tDCS to the left DLPFC had a modulatory effect on memory decay during delay intervals and could enhance memory in young adults; All of these cognitive abilities were also significantly affected by mental fatigue. Studies have shown that tDCS is an effective anti-fatigue measure, and its effect lasts longer than caffeine and is more positive in mood (McIntire et al., 2017). A single blind, randomized trial (Fortes et al., 2022) hint anodal tDCS can relieve mental fatigue of the harmful effects of athletic performance, improve the efficiency of the task. Recently, another form of non-invasive transcranial electrical stimulation tACS has been developed and is increasingly being used to improve aspects related to mental fatigue such as attention and alertness (Fusco et al., 2022; Grover et al., 2023). tACS is a widely used non-invasive method of brain stimulation that involves applying low intensity electrical currents alternately at a prescribed frequency between at least two electrodes on a subject’s scalp (Herzog et al., 2023), usually using current waveforms such as sine, triangle and square waves. Clayton et al. (2019) suggested that α-tACS applied to the partooccipital cortex during a sustained attention task could prevent deterioration of attention to visual attention. A study by Martínez-Pérez et al. (2022) applied 6 and 10 Hz tACS to the right DLPFC during a vigilance task, and their goal was to counteract the typical decrease in vigilance and increase in fatigue usually observed during task time, the results showed that only α frequency (10 Hz) stimulation could improve the performance of the alert executive component.

However, few studies have directly compared the effects of tDCS with those of tACS, which differs from tDCS in that it delivers an alternating current of a specified frequency in a bidirectional manner between the electrodes, whereas tDCS delivers a unidirectional current. tACS likewise has a number of features and methodological considerations similar to those of tDCS, such as the stimulation duration, the site of stimulation, and the current intensity used (Moliadze et al., 2010; Moreira et al., 2022; De Guzman et al., 2023; Liu et al., 2023). Kim et al. (2021) directly compared the effects of tDCS and γ-tACS on cognitive enhancement in mild cognitive impairment using the same current intensity and stimulation duration. γ-tACS and tDCS significantly improved cognitive test scores compared to sham stimulation, and the enhancement of γ-tACS was superior to that of conventional tDCS; In terms of working memory, one study (Röhner et al., 2018) compared a visual 2-back task with 6 Hz tACS and conventional tDCS stimulation, and the results elucidated the clear benefits of tACS compared to tDCS. To date, no study has directly compared the effects of tDCS and tACS on relieving mental fatigue in healthy people. This study will compare the intervention effects of tDCS and α-tACS on mental fatigue, and set up a blank control group. The stimulation design, such as electrode position, current intensity and stimulation duration, was kept consistent during the experiment to match the parameters of α-tACS and tDCS.

The above studies mainly used subjective scales and task performance to evaluate the effect of stimulation, and these results may be affected by mental effort or practice effect (Kim et al., 2021). Previous studies have shown that electrophysiological parameters are better indicators to objectively verify the effect of tES on fatigue (Linnhoff et al., 2021). Objective parameters such as EEG and Magnetic resonance imaging can be used to better evaluate the stimulation effect, but these operations are complicated and cannot be collected conveniently. Recently, it has been shown that there is a strong correlation between HRV parameters of ECG and EEG parameters (Wang et al., 2018), which can accurately and quickly identify the degree of mental fatigue in humans (Chen et al., 2023). Huang et al. (2018) used a wearable smart electrocardiogram (ECG) device to detect the likelihood of mental fatigue state and the results indicated that mean NN, PNN50, TP, and LF were the most important HRV metrics for mental fatigue detection. Murugan et al. (2020) detected and analyzed the state of drivers by monitoring their ECG. Thirteen statistically significant HRV features in ECG signals were extracted for mental fatigue classification with 96.6% accuracy. A study (Ni et al., 2022) also used HRV features for mental fatigue detection, demonstrating the possibility of using HRV for fatigue assessment. Butkeviciute et al. (2022) suggested that the greatest differences between states were found in ECG signal parameters (e.g., Q and R wave amplitudes), as well as QT and T intervals. The accuracy of ECG for mental fatigue discrimination was more than 94.5%, suggesting that ECG can be effective in assessing the degree of mental fatigue. Whether mental fatigue improves after tES intervention can be analyzed using these features for fatigue status, so we used ECG signals for an objective assessment of the effectiveness of mental fatigue intervention.

In summary, the present study will compare the intervention effects of tDCS and α-tACS in mental fatigue, and also assess their effects using ECG. We hypothesized that: anodal tDCS and α- tACS at 10 Hz applied to the prefrontal cortex can effectively alleviate the state of mental fatigue, and the effect of α- tACS is superior to that of tDCS, as evidenced by a significant difference in the relevant ECG parameters.

## 2 Materials and methods

### 2.1 Participants

The object of study for 10 (5 male, 5 female) healthy volunteers with a mean height of 169.5 ± 9.13 cm, weight of 61.1 ± 9.12 kg, and a mean age of 23.8 ± 1.98 years. We screened all participants for healthy young adults aged 18–30 years, including those with normal or corrected to normal vision, right-handedness, and those without severe sleep disorders. We excluded volunteers with speech disorders, a history of migraine, headaches, skin conditions, any adverse experience of previous tDCS or tACS, head/metal implants, and any volunteers who had participated in a tDCS or tACS study in the 6 months prior to the current study. We asked all participants to be asked not to take any functional beverages or medications in the week prior to the experiment. And to get at least 8 h of continuous sleep the day before the experiment, and to have a regular routine for the week prior to participating in the experiment. All participants were informed of the experimental procedures in advance and agreed to sign an informed consent form.

### 2.2 Procedure

The study used a blank controlled, repeated measures design, and the sequence of stimulus interventions was balanced across participants. Each participant was asked to participate in three separate experiments. In three experimental sessions, all participants received tDCS, tACS, and blank stimulation, and a minimum of 7 days were required between each trial to reduce the continuation effect (Andrews et al., 2011) of stimulation. Each participant received three interventions on the same working day for three consecutive weeks.

**FIGURE 1:**
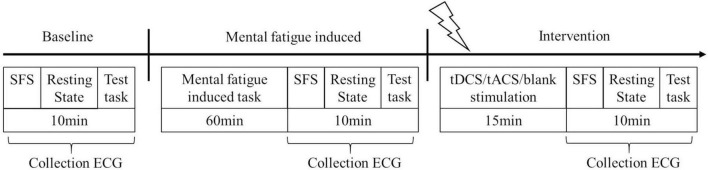
Experimental design.

Each experiment was divided into 3 phases as shown in Figure 1. The experiments lasted a total of 105 min. Phase 1 was the baseline collection phase, which included the collection of participants’ subjective fatigue scale (SFS), a 5 min resting state and a 5 min test task, as well as the collection of participants’ ECG data. Phase 2 was the mental fatigue induction phase, which consisted of a 60 min mental fatigue induction task, collection of the participant’s SFS, a 5 min resting state, and a 5 min test task, along with collection of the participant’s ECG data. Phase 3 was the intervention phase, which consisted of a 15 min intervention (tDCS/tACS/blank), collection of the participant’s SFS, a 5 min resting state and a 5 min test task, along with collection of the participant’s ECG data. According to different experimental stages, they were divided into three different states: pre-fatigue state at baseline, post-fatigue state after fatigue induction, and post-stimulation state after stimulation.

### 2.3 Mental fatigue induction paradigms and test tasks

The mental fatigue-inducing paradigm was chosen to be the TloadDback task, which is a dual task combining the classical N-back working memory updating task and the odd/even decision-making task, and which has been shown to successfully induce fatigue when performed for long periods of time (O’Keeffe et al., 2020; Benkirane et al., 2022). In this experiment, the 2-back+ parity task was used, using the E-prime3.0 design paradigm, in which letters and numbers appeared alternately, with a time interval of 800 ms. If the screen is displayed as a letter: it is necessary to determine whether the current letter is the same as the last letter, press the “H” key when the same letter is the same, and do not react when it is different; If the screen is shown as a number: it is necessary to judge the parity of the current number, press “J” key when it is odd, and press “K” key when it is even. This task was likewise a test task for all three phases of the experiment.

The performance of these tests was assessed by the percentage of correct trials and the average reaction time (Shigihara et al., 2013). Correctness for the test task was calculated as the number of correct trials divided by the total number of trials, and since there was no corresponding reaction time when participants did not respond to key presses, reaction times were averaged by removing the trials in which no keys were pressed.

### 2.4 Subjective fatigue scale

We used the subjective fatigue scale to collect basic information and subjective fatigue levels from participants. The scale was based on the multidimensional fatigue inventory (MFI) (Lim and Son, 2022) and the mental fatigue scale (MFS) (Norlander et al., 2021), and 15 fatigue-related items were selected, ranging from “not at all,” “somewhat obvious,” “generally obvious,” “relatively obvious” to “very obvious,” indicating a gradually deepening degree of correlation, and the scores were “0”–“4”, respectively. Participants were asked to tick the options that corresponded to their situation, and a scale score was calculated based on the scores corresponding to these options, so that they were categorized according to the scale score as normal state “0–15”, mild fatigue “16–30”, moderate fatigue “31–45” and severe fatigue “46–60.”

### 2.5 Transcranial electrical stimulation device

**FIGURE 2:**
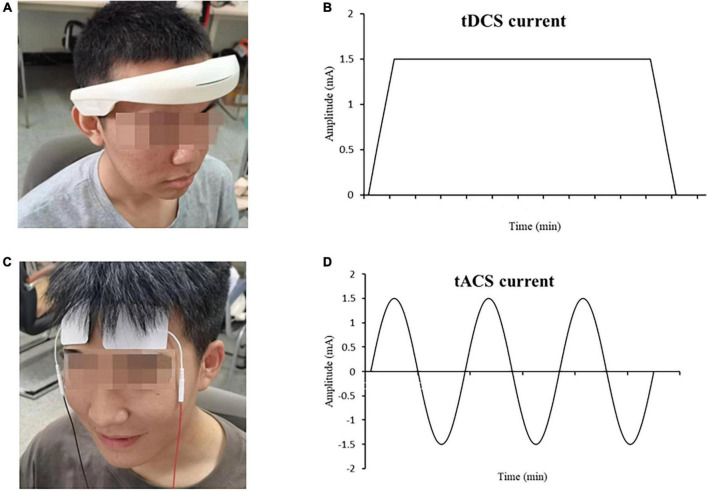
Electrical stimulation device **(A)** tDCS, **(B)** tDCS current intensity trend, **(C)** tACS, **(D)** tACS current intensity trend.

Transcranial direct current stimulation using stimulation device is our independent research and development of the brain stimulator, as shown in Figure 2A. An oval soothing gel patch was used to cover the 5 cm × 5 cm (25 cm^2^) electrode sheet to alleviate the tingling sensation caused by the current. All participants were seated in the same chair for the intervention. Based on the extended International 10–20 system, tDCS interventions were performed in the supraorbital region of the prefrontal lobe, with the anode placed at Fp1 and the cathode at Fp2. The current intensity was 1.5 mA, and the stimulation was sustained for 15 min, consisting of a 30-s rise time at the beginning and a 30-s fall time at the end. As shown in Figure 2B. tACS was stimulated in the right and left prefrontal brain regions (Fp1, Fp2) as shown in Figure 2C. The current intensity was 1.5 mA; the frequency was 10 Hz (using sine-wave alternating current), as shown in Figure 2D; and the stimulation duration was 15 min.

### 2.6 ECG recording and preprocessing

**FIGURE 3:**
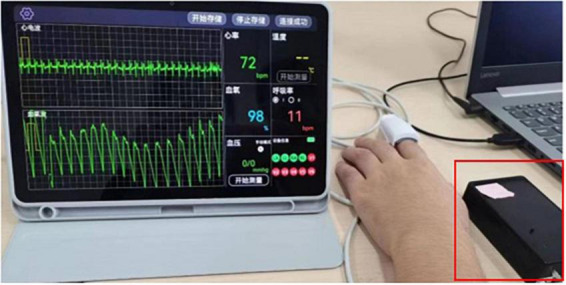
ECG acquisition scene graph.

In this study, ECG signals were acquired using a multiparametric device developed by our own group (see Figure 3), with a sampling rate of 500 Hz. 12 leads were generally used for ECG acquisition, including 6 limb leads (I, II, III, aVR, aVL, aVF) and 6 chest leads (V1∼V6) (Ribeiro et al., 2020). To reduce the complexity of wearing electrodes, six limb leads (I, II, III, aVR, aVL, aVF) out of 12 leads were used in this study, with specific electrode placements in the left upper limb, right upper limb, left lower limb, and right lower limb. The steps of ECG acquisition using the multi-parameter device are as follows: first, wear the ECG electrodes; use Bluetooth to connect to the host computer to control the data transmission; and after successful connection, click on “start storing” to start ECG acquisition. Matlab 2020 software was used to process and analyze the ECG signals. The Pan-Tompkins algorithm (Liu et al., 2017) was used for ECG preprocessing, which was based on the digital analysis of slope, amplitude and width for detection. In addition, the algorithm periodically adjusted the threshold and parameters according to the morphology of QRS complex and the changes of heart rate. After filtering and baseline drift correction, the heart rate variability (HRV) of RR wave interval was extracted. The time domain, frequency domain and nonlinear analysis of HRV series were performed to obtain the time domain features: meanHR, meanRR, SDNN, RMSSD, TINN, PNN50 and SDSD. Frequency domain features: HF, LF, HFn, LFn, LFn/HFn, TP, VLF, and nonlinear features: SD1, SD2, S, VLI, PI and CCM_l, a total of 21 features, and their meanings are shown in Table 1, and the computation of features such as SDNN, RMSSD, PNN50, SDSD, and HFn and LFn are demonstrated in the Table 1.

**TABLE 1:** Meaning of HRV characteristics.

Domain of features	Features	Meaning
Time domain	meanHR	The average heart rate
	meanRR	The mean value of the RR interval
	SDNN	Standard deviation of RR interval SDNN = $\sqrt{\frac{1}{N}\,\sum_{i=1}^{N}{\left(R\,R_{i}-\bar{R\,R}\right)}^{2}}$
	RMSSD	Root mean square of the difference between adjacent RR intervals RMSSD = $\sqrt{\frac{1}{N-1}\,\sum_{i=1}^{N-1}{\left(\text{R}\,R_{i+1}-\text{R}\,R_{i}\right)}^{2}}$
	TINN	Describe the RR interphase histogram approximation of triangle bottom width
	PNN50	NN50 as a percentage of the total RR interval PNN50 = $\frac{N\,N\,50}{N}\times 100\%$
	SDSD	The difference between the maximum and minimum values of the RR interval SDSD = $\sqrt{\frac{1}{N-1}\,\sum_{i=1}^{N}{\left(\bar{\text{R}\,R_{i+1}}-\bar{\text{R}\,R_{i}}\right)}^{2}}$
Frequency domain	HF	High frequency: 0.15–0.4 Hz
	LF	Low frequency band: 0.04–0.15 Hz
	HFn	Normalized high frequency power HFn = $\frac{\text{HF}}{T\,P-V\,L\,F}\times 100\%$
	LFn	Normalized low frequency power LFn = $\frac{\text{LF}}{T\,P-V\,L\,F}\times 100\%$
	LFn/HFn	The ratio of LFn to HFn
	TP	Total power
	VLF	Very low frequency band: 0.003–0.04 Hz
Nonlinear	SD1	Poincare scatter plots were fitted to elliptic semiminor axes
	SD2	Poincare scatter plots were fitted to the semi-major axis of the ellipse
	SD1/SD2	Ratio of semi-minor axis to semi-major axis
	S	Poincare scatterplot fitting ellipse area
	VLI	Vector length exponent
	PI	The percentage of Porta index
	CCM_I	The middle phase sequence correlation between multiple RR

These features above have been shown to have great relevance in fatigue recognition (Huang et al., 2018; Murugan et al., 2020; Butkeviciute et al., 2022; Ni et al., 2022; Chen et al., 2023) and can accurately identify the mental fatigue state of the participants, and whether mental fatigue improves after tES intervention can be analyzed in terms of fatigue state using these features, so we used these features for the assessment of the effect of tES intervention.

### 2.7 Statistical analyses

All statistical analyses were done using SPSS 26 software. For all analyses, *P* < 0.05 was considered statistically significant. Prior to statistical analyses, data were tested for normal distribution using the Shapiro–Wilk test and for homogeneity of variance test using the Levenes test, with *P* > 0.05 considered to be consistent with normal distribution or homogeneity of variance.

Subjective fatigue scale scores were compared between different states (pre-fatigue, post-fatigue, and post-stimulation) in each of the three groups (tDCS group, tACS group, and blank group) using one-way ANOVA, and if *P* < 0.05 then two-by-two tests were performed using Bonferroni post-hoc test. Paired *t*-tests were used to check whether there was statistical significance in performance between the three different states (pre-fatigue, post-fatigue, and post-stimulation) in the three groups. Independent samples *t*-tests were used to compare whether there was statistical significance in ECG features before and after the intervention within each group.

## 3 Results

### 3.1 Baseline level

In this study, a repeated measures ANOVA was performed on participants’ baseline levels for the three experiments. The results did not show any significant differences between the three interventions (tDCS, tACS, and blank group) in subjective fatigue scale scores (*F* = 0.491, *p* = 0.617), response time of test tasks (*F* = 1.481, *p* = 0.245), and accuracy of test tasks (*F* = 0.082, *p* = 0.921).

### 3.2 Subjective fatigue scale analysis

Higher scores on the subjective fatigue scale corresponded to deeper fatigue. One-way ANOVA was performed on the different states (pre-fatigue, post-fatigue, and post-stimulation) in each of the three groups (tDCS group, tACS group, and blank group). Significant differences were found in the blank group (*F* = 12.49, *p* < 0.001), tDCS group (*F* = 19.87, *p* < 0.001) and tACS group (*F* = 18.21, *p* < 0.001). Two-by-two comparisons of arbitrary states were then performed using the Bonferroni post-hoc test.

**FIGURE 4:**
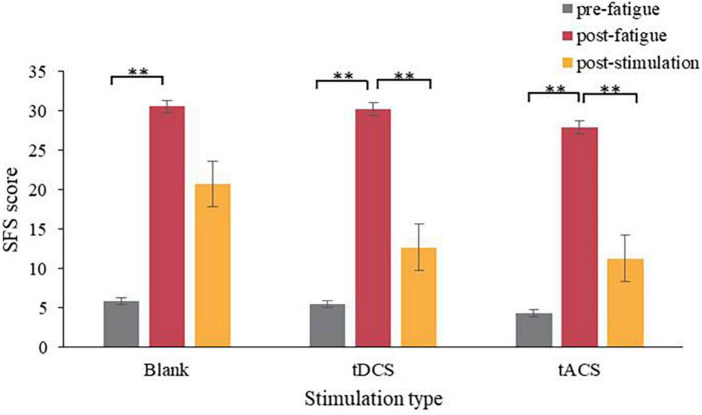
Statistical chart of scale scores. **Means significantly different (*P* < 0.05).

Figure 4 provides a comparison of the subjects’ before and after fatigue (pre-fatigue vs. post-fatigue) and before and after the intervention (post-fatigue vs. post-stimulation). Firstly, it is noticed that there is a significant difference between before and after fatigue in the blank, tDCS and tACS groups (*p* < 0.001) and that the scale scores of all three groups increased significantly after fatigue. It indicates that the subjective fatigue level of the subjects was significantly deepened after the TloadDback task induced fatigue in subjective evaluation. Secondly, before and after the intervention, there was a slow downward trend in the scores of the blank group, which was not significant (*P* = 0.18), and a significant downward trend in the scores of both the tDCS and tACS groups (*P* < 0.001). It indicates that both the tDCS and tACS groups had an effect on the fatigue relief of the participants in terms of subjective evaluation.

**FIGURE 5:**
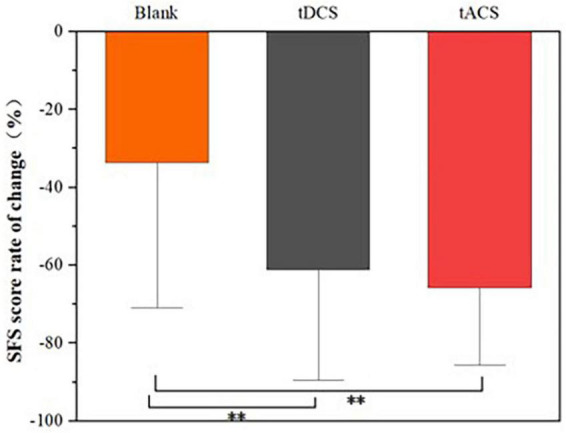
Comparison of relative rates of change between groups. **Means significantly different (*P* < 0.05).

**FIGURE 6:**
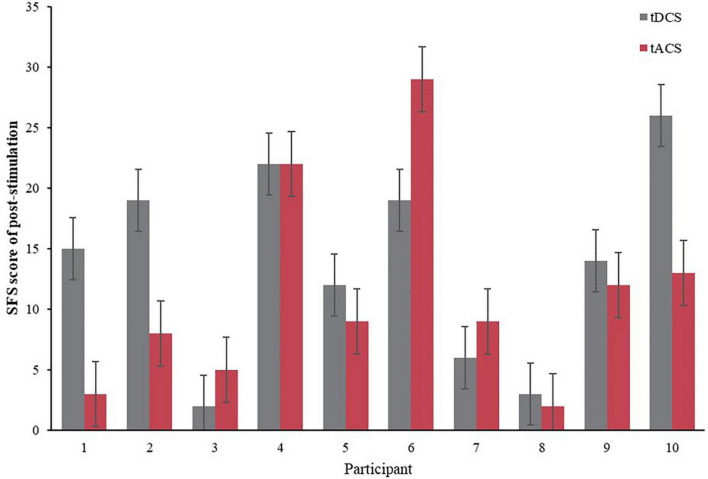
Comparison of SFS scores of tDCS and tACS after intervention.

In order to compare the effects of the tDCS and tACS groups, the relative rates of change (see Equation 1) of the three groups before and after the intervention (post-fatigue vs. post-stimulation) were compared. The relative rates of change were obtained by using the post-stimulation values subtracted from the post-fatigue values, and finally compared to the post-fatigue values, resulting in a value whose greater absolute value indicated a better stimulation effect. Figure 5 compares the relative rates of change between the two groups, and it can be seen that the tACS group had the greatest downward trend compared to the blank and tDCS groups. Further, Figure 6 demonstrates the difference in scale scores for each subject after the tDCS and tACS group interventions, respectively. It is worth noting that in 6 out of 10 subjects, the tACS intervention provided better relief than the tDCS intervention. These results may imply that the tACS group had a more positive impact on fatigue relief than the tDCS group.


$$\text{Rate of change}=\frac{\left({\text{value}}_{\text{post-stimulation}}-{\text{value}}_{\text{post-fatigue}}\right)}{{\text{value}}_{\text{post-fatigue}}}\times 100\%$$

### 3.3 Test task performance analysis

Performance on the test task consisted of the mean reaction time (RT) and the correct rate (ACC) of the task. Since the time interval between the appearance of each letter or number in the test task was 800 ms, the difference in RT was too small to reflect the variation in participants’ response rate. The following analysis then analyses only the ACC of performance.

Table 2 exhibits the results of paired *t*-tests for ACC before and after fatigue (pre-fatigue vs. post-fatigue) for the three groups, the ACC of the blank (*t* = 5.882, *P* < 0.001), tDCS (*t* = 3.860, *P* < 0.05), and tACS (*t* = 3.448, *P* < 0.05) groups decreased significantly. That is, the correctness of participants’ responses after the TloadDback task induced fatigue decreased.

**TABLE 2:** Comparison of accuracy (pre-fatigue vs. post-fatigue).

Group	Pre-fatigue	Post-fatigue	*t*	*P*
Blank	90.21 ± 5.60	84.68 ± 8.10	5.882	**0.000**
tDCS	89.00 ± 5.80	82.04 ± 8.50	3.860	**0.004**
tACS	89.56 ± 8.20	83.28 ± 9.70	3.448	**0.007**

Data are expressed as mean ± standard deviation. Bold fonts indicate significant differences in the data.

Table 3 illustrates the effect of the intervention in the three groups, after performing paired *t*-tests on the ACC before and after intervention (post-fatigue vs. post-stimulation) for each of the three groups, the ACC of the blank group had a weak upward trend but was not significant (*t* = −1.936, *P* > 0.05) after the intervention, whereas the ACC of the tDCS (*t* = −3.093, *P* < 0.05) and tACS (*t* = −3.026, *P* < 0.05) groups increased significantly after the intervention. It means that after electrical stimulation, both tDCS and tACS groups enhanced the correctness of the participants.

**TABLE 3:** Comparison of accuracy (post-fatigue vs. post-stimulation).

Group	Post-fatigue	Post-stimulation	*t*	*P*
Blank	84.68 ± 8.10	86.76 ± 7.40	−1.936	0.085
tDCS	82.04 ± 8.50	88.44 ± 7.00	−3.093	**0.013**
tACS	83.28 ± 9.70	88.60 ± 7.10	−3.026	**0.014**

Data are expressed as mean ± standard deviation. Bold fonts indicate significant differences in the data.

**FIGURE 7:**
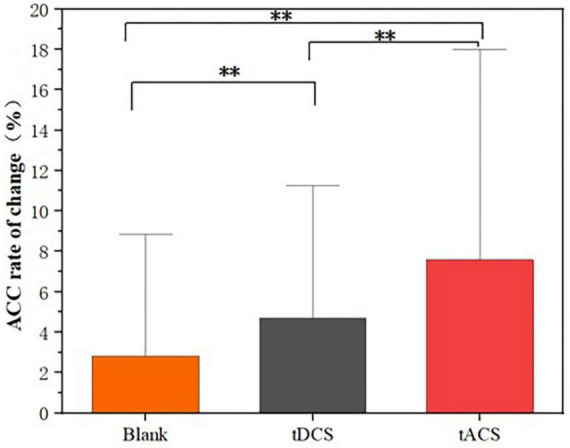
Comparison of relative rates of change. **Means significantly different (*P* < 0.05).

**FIGURE 8:**
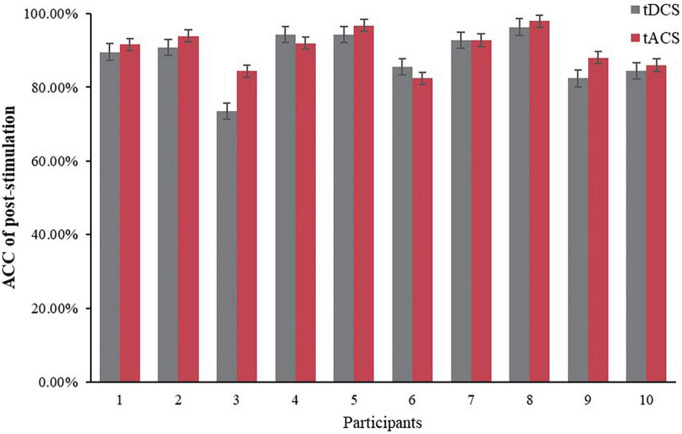
ACC comparison of tDCS and tACS after intervention.

Figure 7 exhibits a comparison of the effects of the tDCS and tACS groups, that is, the relative rates of change (Equation 1) of the three groups before and after the intervention (post-fatigue vs. post-stimulation) were compared, the rate of change rose in the tACS group was greater than that in the tDCS and blank groups. Further, Figure 8 presents a comparison of ACC after tDCS and tACS interventions for the 10 subjects, respectively. It is noteworthy that 8 subjects had greater rate of correctness in the tACS group than in the tDCS group. This indicates a greater improvement in correct rate with tACS than with tDCS.

### 3.4 ECG analysis

Table 4 presents the trends and statistical information of 16 parameters of ECG, including time domain features meanHR, meanRR, SDNN, RMSSD and SDSD, frequency domain features HF, LF, HFn, TP, VLF, and nonlinear features SD1, SD2, S, VLI, and PI, after performing fatigue-inducing tasks in the blank group, tDCS group, and tACS group. Among them, meanHR and LFn are in a decreasing trend and other features are in an increasing trend. The analysis of fatigue level can be carried out using these parameters, and it can be shown that fatigue was successfully induced in all three groups.

**TABLE 4:** Rate of change of HRV features (pre-fatigue vs. post-fatigue).

Feature	meanHR	meanRR	SDNN	RMSSD	PNN50	SDSD	HF	LF	HFn	LFn	TP	VLF	SD1	SD2	S	VLI	PI
tDCS	-0.81	1.43	18.99	19.62	62.99	19.63	44.31	45.23	0.81	-0.28	54.67	67.92	19.63	18.77	50.60	18.78	3.59
Blank	-7.87	9.46	12.97	29.62	67.68	29.79	37.22	17.55	7.77	-3.35	17.08	5.27	29.79	11.08	46.40	11.06	1.81
tACS	-3.38	3.36	12.55	11.57	53.86	11.57	44.77	19.91	6.72	-2.94	21.46	13.00	11.57	12.75	20.16	13.14	3.35
F	4.174	3.769	0.019	0.617	0.076	0.63	0.126	0.412	1.127	0.105	0.485	0.492	0.63	0.057	0.323	0.06	0.598
*P*	**0**.**026**	**0**.**036**	0.981	0.547	0.967	0.54	0.882	0.667	0.339	0.985	0.621	0.617	0.54	0.945	0.747	0.942	0.557

Unit, “%”; “−” indicates a downward trend, and others indicate an upward trend. Significant difference is represented by “*P*,” bold fonts indicate significant differences (*P* < 0.05) in the data.

Table 5 demonstrates the results of independent samples *t*-tests or Mann–Whitney U-tests performed before and after the intervention (post-fatigue vs. post-stimulation) for the three groups. There were no significant difference between the characteristics of the blank group (*P* > 0.05) before and after the intervention, only TINN characteristics of the tDCS group (*P* = 0.046) had a significant difference, and the 11 characteristics of the tACS group (*P* < 0.05), including SDNN, LF, HFn, LFn, LFn, LFn/HFn, TP, VLF, SD2, SD1/SD2, VLI, and CCM_l, had a significant differences. Among them, SDNN (*t* = −3.241, *P* = 0.002), LF (*t* = −3.511, *P* = 0.001), LFn (*t* = −3.122, *P* = 0.002), TP (*t* = −2.706, *P* = 0.008), VLF (*t* = −3.002, *P* = 0.004), SD2 (*t* = −3.594, *P* = 0.001) and VLI (*t* = −3.564, *P* = 0.001) showed an increasing trend after the intervention, while HFn (*t* = 3.122, *P* = 0.002), SD1/SD2 (*t* = 3.158, *P* = 0.002) and CCM_1 (*t* = 3.106, *P* = 0.003) showed a decreasing trend. The tDCS group, although there was no significant difference in these characteristics of SDNN, LF, HFn, LFn, LFn, LFn/HFn, TP, VLF, SD2, SD1/SD2, VLI, and CCM_l, had more or less the same trend of change. This means that the tDCS and tACS groups have a positive effect on these features and the tACS group has a significant effect.

**TABLE 5:** Three groups of ECG analysis (post-fatigue vs. post-stimulation).

Feature	Blank		tDCS		tACS	
	$\bar{\text{X}}$	*P*	$\bar{\text{X}}$	*P*	$\bar{\text{X}}$	*P*
meanHR	0.814	0.647	-0.403	0.824	0.545	0.657
meanRR	-10.903	0.577	5.682	0.808	-6.356	0.641
SDNN	-2.361	0.725	-7.501	0.290	-9.865	**0.002**
RMSSD	-2.166	0.615	-4.769	0.562	-0.005	0.999
TINN	3.033	0.853	-31.168	**0.046**	-17.230	0.090
PNN50	-0.001	0.963	-0.012	0.792	0.007	0.817
SDSD	-2.172	0.615	-4.775	0.562	-0.035	0.990
HF	-126.742	0.625	-597.922	0.371	-63.690	0.747
LF	-296.572	0.684	-958.115	0.205	-964.933	**0.001**
HFn	0.804	0.773	0.834	0.803	9.402	**0.002**
LFn	-0.804	0.773	-0.834	0.803	-9.402	**0.002**
LFn/HFn	0.499	0.576	0.007	0.992	-2.248	**0.005**
TP	-1079.290	0.567	-2634.019	0.203	-1801.064	**0.008**
VLF	-655.976	0.483	-1077.983	0.195	-899.821	**0.004**
SD1	-1.536	0.615	-3.377	0.562	-0.024	0.990
SD2	-2.988	0.742	-10.261	0.235	-14.694	**0.001**
SD1/SD2	-0.007	0.640	0.008	0.792	0.068	**0.002**
S	-1300.304	0.541	-3454.941	0.307	-1311.845	0.098
VLI	-2.997	0.741	-10.319	0.234	-14.641	**0.001**
PI	-0.208	0.722	-0.501	0.541	-0.926	0.335
CCM_l	-0.003	0.664	0.003	0.832	0.039	**0.003**

$\bar{\text{X}}$, mean difference before and after intervention, significant difference is represented by “*P*,” bold fonts indicate significant differences (*P* < 0.05) in the data.

In summary, mental fatigue affects a number of ECG parameters. α-tACS at 10 Hz counteracted the development of mental fatigue and significantly affected changes in ECG parameters compared with the blank and tDCS groups.

## 4 Discussion

The purpose of this study was to compare the mitigating effects of anodic tDCS and α-tACS on mental fatigue. To the best of our knowledge, this is the first study to directly compare the mental fatigue-relieving effects of tDCS and tACS in healthy youth. The results generally indicate that both stimulus types can achieve relief of mental fatigue. Quantitative and qualitative assessments were performed using the subjective fatigue scale, test task performance, and HRV characteristics of ECG signals, respectively. It can be seen that the rate of change of decrease in subjective fatigue scale scores and the rate of change of increase in test task ACC were greater in the tACS group than in the tDCS group; and eleven of the HRV features in the tACS group were significantly different after the intervention, which was much more than the number of features in the tDCS group. These indicated that tACS was more effective than tDCS.

Despite the growing interest in transcranial electrical stimulation for fatigue relief, results in the literature have been mixed. The present study indicates that both tACS and tDCS can achieve relief of mental fatigue, which is consistent with the findings of Fortes et al. (2022). Whereas some findings indicated that tES did not have beneficial effects on fatigue, in comparison to the present study, Moreira et al.’s (2022) study used a different stimulation time and a different group of subjects, whereas De Guzman et al. (2023) stimulated the area of the primary motor cortex. These differences in results may then be due to different stimulus intensities, durations and subject populations. Our study indicated a somewhat better effect of tACS, which is consistent with the report of Martínez-Pérez et al. (2022) that the use of tACS at 10 Hz can enhance alpha wave activity in frontal regions. Improved cognitive control and attention levels. We used ECG for mental fatigue discrimination to enable assessment of tES, which is more convenient to acquire and easier to obtain than EEG signals used by Liu et al. (2023) and magnetoencephalography used by Kasten et al. (2018). The results of the study indicated that the time-domain features SDNN, frequency-domain features LF, HFn, LFn, LFn, LFn/HFn, TP, VLF, and nonlinear features SD2, SD1/SD2, VLI, and CCM_l were significantly different (*p* < 0.05) before and after the intervention. There is early evidence that these features can be used to assess mental fatigue. Hao et al. (2022) indicated that HR, RRn, SDNN, RMSSD, pNN50, CV, HF, SD1, and SD2 parameters in ECG variability are useful sensitivity parameters to reliably detect the presence of mental load in personnel. Similarly it has been shown that the probability density functions of SDNN, SDSD, VLF, HFnorm and LF/HF are significantly different in both non-fatigued and fatigued states (Gao et al., 2022). Anwer et al. (2023) on the other hand verified that both linear and non-linear HRV analyses can be used to accurately detect and classify fatigue levels. These demonstrate that ECG can be effective in assessing the level of mental fatigue and in evaluating the effectiveness of tES.

Research has shown that the alpha wave is a type of brain wave associated with relaxation, concentration, and cognitive control, among other things. After long hours of work or study, people’s alpha wave activity decreases, which causes mental fatigue (Li et al., 2023). Our use of α-tACS at 10 Hz in the prefrontal cortex enhances α-wave activity in the frontal region and alleviates mental fatigue. HRV is the change in heartbeat interval. Under normal conditions, heartbeat intervals change due to sympathetic and parasympathetic regulation. HRV is a physiological indicator of the functional state of the autonomic nervous system and has an important role in assessing mental fatigue (Hao et al., 2022). In the present study, intervention for mental fatigue using α-tACS at 10 Hz increased the high-frequency component of HRV, which may reflect enhanced regulation of the autonomic nervous system. The high-frequency component is often considered an indicator of parasympathetic influence, and its increase reflects a change in the balance between sympathetic and parasympathetic nerves. tACS promotes synchronized activity in cortical areas of the brain. This synchronized activity may influence the regulation of the autonomic nervous system through a neurofeedback mechanism and increase the high-frequency component of HRV. Meanwhile, we found that tACS significantly enhances the total power of HRV, which reflects a rise in the sympathetic activity and the overall activity of the autonomic nervous system, which play a dominant role, and the degree of mental fatigue will be reduced. The physiological mechanism of action for the significant effect of α-tACS at 10 Hz on HRV characteristics in the present study may involve the influence of autonomic nervous system regulation, neurofeedback mechanisms, and other aspects. This finding provides some insight into the use of α-tACS to modulate HRV features.

For the HRV features with significant differences obtained in this study, we can use these significant features to assess the effectiveness of mental fatigue interventions and make theoretical support for the use of ECG signals to detect the effects of tES. Of course, we can also use these features for further accurate recognition of mental fatigue. That is, automated identification and assessment of mental fatigue using some machine learning or deep learning models for these significant features. Currently, our study uses offline data for analysis. Then, furthermore, we can establish a multilevel mental fatigue recognition model based on ECG features, collect the ECG signals of the subjects in real time and output the mental fatigue level in real time. Then, according to the obtained results, a suitable intervention programme is given, and the mental fatigue level of the subject is output in real time after the intervention. The combination of real-time mental fatigue detection based on ECG signals and intervention measures forms a closed-loop system. Through this closed-loop system, we can monitor the changes of ECG characteristics and mental fatigue level in real time, evaluate the effectiveness of the intervention in a timely manner, and adjust and optimize the guiding therapeutic plan in a timely manner. This will facilitate the researcher to control the stimulation duration and intensity of the transcranial electrical stimulation device individually, and to be able to determine the best personalized intervention plan for the subjects.

## 5 Conclusion

This study compared the intervention effects of anodal tDCS and α-tACS in mental fatigue. The analysis of subjective fatigue scales, test task performance and ECG features concluded that anodal tDCS and α-tACS significantly reduced subjective fatigue and increased correctness on the test task, while the degree of change was greater in the α-tACS compared to the tDCS and blank groups. For the HRV features extracted from ECG signals, there were no significant differences in any of the features in the blank group. tDCS group had significant differences only in the feature TINN. Eleven features such as SDNN, LF, HFn, LFn, LFn, LFn/HFn, TP, VLF, SD2, SD1/SD2, VLI, and CCM_l were significantly different (*P* < 0.05) before and after intervention in the tACS group. This indicated that both tDCS and α-tACS could effectively alleviate mental fatigue, and α-tACS was more effective than tDCS. This study provides theoretical support that tDCS and α-tACS have a relieving effect on mental fatigue. It also validates the use of ECG as an objective assessment tool for tES. This finding provides some insights into the use of α-tACS to modulate HRV characteristics to improve mental fatigue.

In future work, more subjects of different age groups and physical health levels will be included to improve the reliability of tES used to relieve mental fatigue. Secondly, we selected a limited number of stimulation parameters and a short stimulation duration for tES, and in the future, more stimulation parameters should be selected to compare their effects, expand the scope of the study, and select the optimal combination of protocols for testing. Finally, the use of other physiological signals and imaging techniques will be considered to evaluate the intervention effects.

## Data availability statement

The raw data supporting the conclusions of this article will be made available by the authors, without undue reservation.

## Ethics statement

The studies involving humans were approved by the Ethics Committee of Biomedical Research involving Human Beings, Shandong Provincial Hospital. The studies were conducted in accordance with the local legislation and institutional requirements. The participants provided their written informed consent to participate in this study. Written informed consent was obtained from the individual(s) for the publication of any potentially identifiable images or data included in this article.

## Author contributions

RC: Methodology, Writing – review & editing. LH: Validation, Writing – original draft. RW: Software, Writing – review & editing. JF: Investigation, Writing – review & editing. HW: Funding acquisition, Writing – review & editing. JW: Project administration, Supervision, Writing – review & editing.

## References

[B1] Abdollahzade Z. Hadian M. R. Khanmohammadi R. Talebian S. (2023). Efficacy of stretching exercises versus transcranial direct current stimulation (tDCS) on task performance, kinematic and electroencephalography (EEG) spectrum in subjects with slump posture: A study protocol. *Trials* 24:351. 10.1186/s13063-023-07359-0 37221565 PMC10207831

[B2] Andrews S. C. Hoy K. E. Enticott P. G. Daskalakis Z. J. Fitzgerald P. B. (2011). Improving working memory: The effect of combining cognitive activity and anodal transcranial direct current stimulation to the left dorsolateral prefrontal cortex. *Brain Stimul.* 4 84–89. 10.1016/j.brs.2010.06.004 21511208

[B3] Antal A. Alekseichuk I. Bikson M. Brockmöller J. Brunoni A. Chen R. (2017). Low intensity transcranial electric stimulation: Safety, ethical, legal regulatory and application guidelines. *Clin. Neurophysiol.* 128 1774–1809. 10.1016/j.clinph.2017.06.001 28709880 PMC5985830

[B4] Anwer S. Li H. Umer W. Antwi-Afari M. Mehmood I. Yu Y. (2023). Identification and classification of physical fatigue in construction workers using linear and nonlinear heart rate variability measurements. *J. Construct. Eng. Manag.* 149:13100. 10.1061/JCEMD4.COENG-13100

[B5] Benkirane O. Delwiche B. Mairesse O. Peigneux P. (2022). Impact of Sleep Fragmentation on Cognition and Fatigue. *Int. J. Environ. Res. Public Health* 19:15485. 10.3390/ijerph192315485 36497559 PMC9740245

[B6] Butkeviciute E. Michalkovic A. Bikulciene L. (2022). ECG signal features classification for the mental fatigue recognition. *Mathematics* 10:10183395. 10.3390/math10183395

[B7] Chen R. Wang R. Fei J. Huang L. Wang J. (2023). Quantitative identification of daily mental fatigue levels based onmultimodal parameters. *Rev. Sci. Instr.* 9:312. 10.1063/5.0162312 37695118

[B8] Clayton M. S. Yeung N. Cohen Kadosh R. (2019). Electrical stimulation of alpha oscillations stabilizes performance on visual attention tasks. *J. Exp. Psychol. Gen.* 148 203–220. 10.1037/xge0000502 30421943

[B9] Dai J. Wang H. Yang L. Wang C. Cheng S. Zhang T. (2022). The neuroelectrophysiological and behavioral effects of transcranial direct current stimulation on executive vigilance under a continuous monotonous condition. *Front. Neurosci.* 16:910457. 10.3389/fnins.2022.910457 36161182 PMC9489920

[B10] De Guzman K. A. Young R. J. Contini V. Clinton E. Hitchcock A. Riley Z. A. (2023). The influence of transcranial alternating current stimulation on fatigue resistance. *Brain Sci.* 13:1225. 10.3390/brainsci13081225 37626581 PMC10452200

[B11] Fernández A. Cid-Fernández S. Díaz F. (2021). Transcranial direct current stimulation (tDCS). An effective tool for improving episodic memory in young people? *Ann. Psychol.* 37 468–477. 10.6018/analesps.368341

[B12] Fortes L. Faro H. de Lima-Junior D. Albuquerque M. Ferreira M. (2022). Non-invasive brain stimulation over the orbital prefrontal cortex maintains endurance performance in mentally fatigued swimmers. *Physiol. Behav.* 250:113783. 10.1016/j.physbeh.2022.113783 35331714

[B13] Fusco G. Fusaro M. Aglioti S. (2022). Midfrontal-occipital θ-tACS modulates cognitive conflicts related to bodily stimuli. *Soc. Cogn. Affect. Neurosci.* 17 91–100. 10.1093/scan/nsaa125 33448297 PMC8824600

[B14] Gao R. Yan H. Duan J. Gao Y. Cao C. Li L. (2022). Study on the nonfatigue and fatigue states of orchard workers based on electrocardiogram signal analysis. *Sci. Rep.* 12:4858. 10.1038/s41598-022-08705-z 35318355 PMC8940960

[B15] Gao S. Zhou K. Zhang J. Cheng Y. Mao S. (2023). Effects of background music on mental fatigue in steady-state visually evoked potential-based BCIs. *Healthcare* 11:1014. 10.3390/healthcare11071014 37046941 PMC10094051

[B16] Gecaite-Stonciene J. Hughes B. Burkauskas J. Bunevicius A. Kazukauskiene N. van Houtum L. (2021). Fatigue is associated with diminished cardiovascular response to anticipatory stress in patients with coronary artery disease. *Front. Physiol.* 12:692098. 10.3389/fphys.2021.692098 34483954 PMC8416171

[B17] Grover S. Fayzullina R. Bullard B. Levina V. Reinhart R. M. G. (2023). A meta-analysis suggests that tACS improves cognition in healthy, aging, and psychiatric populations. *Sci. Transl.* 15:abo2044. 10.1126/scitranslmed.abo2044 37224229 PMC10860714

[B18] Hao T. Zheng X. Wang H. Xu K. Chen S. (2022). Linear and nonlinear analyses of heart rate variability signals under mental load. *Biomed. Signal Process. Control* 77:103758. 10.1016/j.bspc.2022.103758

[B19] Herden L. Weissert R. (2020). The effect of coffee and caffeine consumption on patients with multiple sclerosis-related fatigue. *Nutrients* 12:2262. 10.3390/nu12082262 32731633 PMC7468779

[B20] Herzog R. Bolte C. Radecke J. O. von Möller K. Lencer R. Tzvi E. (2023). Neuronavigated cerebellar 50 hz tACS: Attenuation of stimulation effects by motor sequence learning. *Biomedicines* 11:2218. 10.3390/biomedicines11082218 37626715 PMC10452137

[B21] Holgado D. Troya E. Perales J. Vadillo M. Sanabria D. (2021). Does mental fatigue impair physical performance? A replication study. *Eur. J. Sport Sci.* 21 762–770. 10.1080/17461391.2020.1781265 32519588

[B22] Huang S. Li J. Zhang P. Zhang W. (2018). Detection of mental fatigue state with wearable ECG devices. *Int. J. Med. Inf.* 119 39–46. 10.1016/j.ijmedinf.2018.08.010 30342684

[B23] Kasten F. Maess B. Herrmann C. (2018). Facilitated event-related power modulations during transcranial alternating current stimulation (tACS) revealed by concurrent tACS-MEG. *ENEURO* 5 1–15. 10.1523/ENEURO.0069-18.2018 30073188 PMC6070188

[B24] Kim J. Kim H. Jeong H. Roh D. Kim D. (2021). TACS as a promising therapeutic option for improving cognitive function in mild cognitive impairment: A direct comparison between tACS and tDCS. *J. Psychiatr. Res.* 141 248–256. 10.1016/j.jpsychires.2021.07.012 34256276

[B25] Li G. Zhang L. Zou Y. Ouyang D. Yuan Y. Lian Q. (2023). Driver vigilance detection based on limited EEG signals. *IEEE Sens. J.* 23 13387–13398. 10.1109/JSEN.2023.3273556

[B26] Lim E. Son C. (2022). Comparison of assessment scores for fatigue between multidimensional fatigue inventory (MFI-K) and modified chalder fatigue scale (mKCFQ). *J. Transl. Med.* 20:8. 10.1186/s12967-021-03219-0 34980164 PMC8722196

[B27] Linnhoff S. Wolter-Weging J. Zaehle T. (2021). Objective electrophysiological fatigability markers and their modulation through tDCS. *Clin. Neurophysiol.* 132 1721–1732. 10.1016/j.clinph.2021.02.391 33867262

[B28] Liu F. Wei S. Li Y. Jiang X. Zhang Z. Zhang L. (2017). The accuracy on the common pan-tompkins based QRS detection methods through low-quality electrocardiogram database. *J. Med. Imaging Health Inf.* 7 1039–1043. 10.1166/jmihi.2017.2134

[B29] Liu Y. Liu Q. Zhao J. Leng X. Han J. Xia F. (2023). Anodal transcranial direct current stimulation (tDCS) over the left dorsolateral prefrontal cortex improves attentional control in chronically stressed adults. *Front. Neurosci.* 17:1182728. 10.3389/fnins.2023.1182728 37397442 PMC10309114

[B30] Martínez-Pérez V. Tortajada M. Palmero L. B. Campoy G. Fuentes L. (2022). Effects of transcranial alternating current stimulation over right-DLPFC on vigilance tasks depend on the arousal level. *Sci. Rep.* 12:547. 10.1038/s41598-021-04607-8 35017631 PMC8752588

[B31] McIntire L. McKinley R. Nelson J. Goodyear C. (2017). Transcranial direct current stimulation versus caffeine as a fatigue countermeasure. *Brain Stimul.* 10 1070–1078. 10.1016/j.brs.2017.08.005 28851554

[B32] Moliadze V. Antal A. Paulus W. (2010). Boosting brain excitability by transcranial high frequency stimulation in the ripple range. *J. Physiol.* 588 4891–4904. 10.1113/jphysiol.2010.196998 20962008 PMC3036186

[B33] Moreira A. Moscaleski L. Machado D. Bikson M. Unal G. Bradley P. (2022). Transcranial direct current stimulation during a prolonged cognitive task: The effect on cognitive and shooting performances in professional female basketball players. *Ergonomics* 66 492–505. 10.1080/00140139.2022.2096262 35766283

[B34] Murugan S. Selvaraj J. Sahayadhas A. (2020). Detection and analysis: Driver state with electrocardiogram (ECG). *Phys. Eng. Sci. Med.* 43 525–537. 10.1007/s13246-020-00853-8 32524437

[B35] Ni Z. Sun F. Li Y. (2022). Heart rate variability-based subjective physical fatigue assessment. *Sensors* 22:3199. 10.3390/s22093199 35590889 PMC9100264

[B36] Norlander A. Lindgren I. Pessah-Rasmussen H. Gard G. Brogårdh C. (2021). Fatigue in men and women who have returned to work after stroke: Assessed with the fatigue severity scale and mental fatigue scale. *J. Rehabil. Med.* 53:9. 10.2340/16501977-2863 34383959 PMC8638741

[B37] O’Keeffe K. Hodder S. Lloyd A. A. (2020). comparison of methods used for inducing mental fatigue in performance research: Individualised, dual-task and short duration cognitive tests are most effective. *Ergonomics* 63 1–12. 10.1080/00140139.2019.1687940 31680632

[B38] Ribeiro A. Ribeiro M. Paixao G. Oliveira D. Gomes P. Canazart J. (2020). Automatic diagnosis of the 12-lead ECG using a deep neural network. *Nat. Commun.* 11:1760. 10.1038/s41467-020-15432-4 32273514 PMC7145824

[B39] Röhner F. Breitling C. Rufener K. Heinze H. Hinrichs H. Krauel K. (2018). Modulation of working memory using transcranial electrical stimulation: A direct comparison between TACS and TDCS. *Front. Neurosci.* 12:761. 10.3389/fnins.2018.00761 30405341 PMC6206050

[B40] Sehm B. Kipping J. A. Schaefer A. Villringer A. Ragert P. A. (2013). comparison between uni- and bilateral tDCS effects on functional connectivity of the human motor cortex. *Front. Hum. Neurosci.* 7:183. 10.3389/fnhum.2013.00183 23675337 PMC3646257

[B41] Shigihara Y. Tanaka M. Ishii A. Kanai E. Funakura M. Watanabe Y. (2013). Two types of mental fatigue affect spontaneous oscillatory brain activities in different ways. *Behav. Brain* 9:2. 10.1186/1744-9081-9-2 23305089 PMC3562167

[B42] Van Cutsem J. Marcora S. De Pauw K. Bailey S. Meeusen R. Roelands B. (2017). The effects of mental fatigue on physical performance: A systematic review. *Sports Med.* 47 1569–1588. 10.1007/s40279-016-0672-0 28044281

[B43] Wang F. Wang H. Fu R. (2018). Real-time ECG-based detection of fatigue driving using sample entropy. *Entropy* 20:196. 10.3390/e20030196 33265287 PMC7512712

[B44] Wang Y. Wang Q. Wang L. Li F. Weschler L. Huang J. (2023). Potential benefits of short-term indoor exposure to sweet orange essential oil for relaxation during mental work breaks. *J. Build. Eng.* 78 2352–7102. 10.1016/j.jobe.2023.107602

